# Immunological Cross-Reactivity of Proteins Extracted from the Oomycete *Pythium insidiosum* and the Fungus *Basidiobolus ranarum* Compromises the Detection Specificity of Immunodiagnostic Assays for Pythiosis

**DOI:** 10.3390/jof7060474

**Published:** 2021-06-11

**Authors:** Tiwa Rotchanapreeda, Pattarana Sae-Chew, Tassanee Lohnoo, Wanta Yingyong, Thidarat Rujirawat, Yothin Kumsang, Penpan Payattikul, Chalisa Jaturapaktrarak, Akarin Intaramat, Watcharapol Pathomsakulwong, Chompoonek Yurayart, Theerapong Krajaejun

**Affiliations:** 1 Department of Pathology, Faculty of Medicine, Ramathibodi Hospital, Mahidol University, Bangkok 10400, Thailand; ai-kaze@hotmail.com; 2 Research Center, Faculty of Medicine, Ramathibodi Hospital, Mahidol University, Bangkok 10400, Thailand; pattarana.sae@mahidol.ac.th (P.S.-C.); tassanee.loh@mahidol.ac.th (T.L.); wanta.yin@mahidol.ac.th (W.Y.); thidarat.ruj@mahidol.ac.th (T.R.); pusjeckchon@hotmail.com (Y.K.); payattikul@yahoo.com (P.P.); chalisa.jat@mahidol.edu (C.J.); 3 Translational Research Unit and Laboratory of Immunology, Chulabhorn Research Institute, Bangkok 10210, Thailand; akarin@cri.or.th; 4 Equine Clinic, Kasetsart University Veterinary Teaching Hospital, Nakhon Pathom 73140, Thailand; watgolf2000@gmail.com; 5 Department of Microbiology and Immunology, Faculty of Veterinary Medicine, Kasetsart University, Bangkok 10900, Thailand; fvetcny@ku.ac.th

**Keywords:** pythiosis, *Pythium insidiosum*, *Basidiobolus ranarum*, cross-reactivity, serodiagnosis

## Abstract

Pythiosis, a life-threatening disease caused by *Pythium insidiosum*, has been increasingly diagnosed worldwide. A recently developed immunochromatographic test (ICT) enables the rapid diagnosis of pythiosis. During the 3-year clinical implementation of ICT in Thailand, we collected the laboratory reports of 38 animals with suspected pythiosis and detected ICT false-positive results in three horses and a dog with basidiobolomycosis. *P. insidiosum* and *Basidiobolus ranarum* cause infections with indistinguishable clinical and microscopic features. This study investigated cross-reactive antibodies by probing *P. insidiosum* and *B. ranarum* crude extracts and cell-free synthesized I06 protein (encoded in *P. insidiosum* genome, not other fungi) against a panel of pythiosis, basidiobolomycosis, rabbit anti-I06 peptide, and control sera by Western blot analyses. ICT false-positive results occurred from the cross-reactivity of anti-*B. ranarum* antibodies to the 15, 50, 60, and 120 kDa proteins of *P. insidiosum*, not double infections caused by both pathogens. Notably, ICT could help to screen pythiosis, and the positive test requires confirmation by culture or molecular method. The detection specificity of ICT requires improvement. The crude extract containing multispecies antigens needs replacement with a refined *P. insidiosum*-specific protein. We proposed that the 55 kDa I06 protein is an excellent candidate for developing a more specific serodiagnostic test for pythiosis.

## 1. Introduction

Detection of the pathogen-specific antibodies in a serum sample facilitates the accurate diagnosis of many infectious diseases. Such an immunological method bypasses the routine, experience-required, and time-consuming microbiological techniques, i.e., culture and morphological examination. Several serodiagnostic assays have been developed for the rapid diagnosis of pythiosis, a life-threatening infectious condition caused by the fungus-like oomycete microorganism *Pythium insidiosum*, in humans and animals worldwide [1,2,3,4,5,6,7,8,9,10,11,12]. These assays include immunodiffusion (ID), Western blot (WB), enzyme-linked immunosorbent assay (ELISA), hemagglutination (HA), and an immunochromatographic test (ICT) [1,2,3,4,5,6,7,8,9]. The principle of these serological assays relies on the specific binding of crude protein extracts (prepared from the in vitro growth of *P. insidiosum*) and their corresponding antibodies to differentiate pythiosis from other infectious diseases [4,13]. Compared to the other assays, ELISA and ICT show relatively higher assay efficiency and are commonly used for detecting the serum anti-*P. insidiosum* antibodies [1,2,4,9]. However, ICT is a more favorable test format because of its short turnaround time (i.e., 30 min) and user-friendly design (does not require any equipment) [1,2,4]. Inaccurate diagnosis could lead to delayed therapy and poor prognosis (i.e., losing an organ or life) in a patient with pythiosis. Rapid ICT result readout prompts a clinician to plan proper treatment for each pythiosis patient, which likely requires radical surgery (i.e., keratoplasty, enucleation, and limb amputation), as the conventional antimicrobial agents usually fail to fight against *P. insidiosum* [9,10,11,12,14,15].

ICT detected the anti-*P. insidiosum* antibodies in the first Thai case of animal pythiosis in 2017 [16]. Since then, the assay has assisted in diagnosing pythiosis in several centers in Thailand, including our allied laboratory at the Faculty of Veterinary Medicine, Kasetsart University. Confirmed cases of animal pythiosis have arisen because healthcare workers are more familiar with the disease, and diagnostic tools (i.e., culture, polymerase chain reaction (PCR), and ICT) have become widely available [2,3,4,6,7,8,9,17,18,19,20]. On several occasions, the antibody detection and the culture identification showed inconsistent findings (i.e., ICT was positive, but an organism other than *P. insidiosum* was isolated), indicating a false-positive serological result. The crude protein extract of *P. insidiosum*, commonly used to develop the serological assays [1,2,3,4,5,6,7,8,9,13], may share antigenic epitopes with another organism. The false-positive serological result could lead to an adverse consequence because the treatment of pythiosis and other mycoses are generally different (i.e., radical surgery for pythiosis and antimicrobial drug administration for other fungal infections).

Here, we collected diagnostic laboratory data (including the culture and PCR reports) of the ICT-tested animals during the past 3 years and identified a fungal organism that might generate cross-reactive antibodies against *P. insidiosum*. In addition, we produced the 55 kDa immunoreactive OPEL-like I06 protein (uniquely presented in the genome of *P. insidiosum* but not the other medically important fungi) using the cell-free protein synthesis platform [21]. The synthesized I06 proteins, together with rabbit anti-I06 peptide antibodies and a panel of pythiosis and control sera, were employed to investigate the immunological cross-reactivity of *P. insidiosum* and another organism. The obtained information on the nature of the *P. insidiosum* antigens could be applied in developing a better serological assay to provide an accurate diagnosis for pythiosis.

## 2. Materials and Methods

### 2.1. Clinical Uses of ICT and Culture Method for Diagnosis of Animal Pythiosis

ICT was implemented to rapidly detect the anti-*P. insidiosum* antibodies in the serum samples of 38 animals with clinically suspected pythiosis at the Department of Microbiology and Immunology, Faculty of Veterinary Medicine, Kasetsart University, Bangkok, Thailand, during the period between August 2017 and January 2021 (Table 1). The assay was performed as previously described [2]. To summarize, an unknown serum, a positive control serum (from a culture-proven case of animal pythiosis), and a negative control serum (from a healthy animal) were diluted (1:5000) in 0.15× phosphate-buffered saline (0.15× PBS; 1.5 mM Na_2_HPO_4_, 0.27 mM KH_2_PO_4_, 20.55 mM NaCl, 0.405 mM KCl; pH 7.4). Each diluted serum sample (100 μL) was tested for anti-*P. insidiosum* antibodies using ICT. Experienced personnel read the ICT result within 30 min.

A fresh, frozen, or formalin-fixed paraffin-embedded (FFPE) tissue from the affected animal (if available) was subjected to identifying a causative agent. An obtained fresh tissue sample was cultured on Sabouraud dextrose (SD) agar and incubated at room temperature for up to 2 weeks. An isolated microorganism was initially identified to the genus or species level, based on the macroscopic and microscopic features. Genomic DNA (gDNA) was extracted from the fungal culture, frozen tissue, or FFPE tissue using the established DNA extraction procedures [20]. The organism was identified at the species level by PCR amplification and the sequencing of the ITS region of rDNA, using the universal fungal primers (ITS1 (5′-TCCGTAGGTGAACCTGCGG-3′) and ITS4 (5′-TCCTCCGCTAATTGATATGC-3′)) and the established protocol [17,22,23]. The resulting ITS sequence was BLAST searched against the NCBI database for nucleotide similarity. The ICT, culture, and molecular assay results obtained during August 2017 and January 2021 were collected for downstream analysis.

### 2.2. Antigen Preparation

*P. insidiosum* strain Pi–S or *Basidiobolus ranarum* strain KU30017.1 maintained on SD agar were subjected to the preparation of culture filtrate antigen (CFA). Several small agar pieces containing the colony of either microorganism were cultured in SD broth and incubated with shaking (50 rpm) at 37 °C for 10 days. The culture medium was filtrated through a Whatman filter paper grade 1 (GE Healthcare, Chicago, IL, USA) and then a 0.22 μm-pore size Durapore filter membrane (Merck, Darmstadt, Germany) to collect CFA before adding the proteinase inhibitors EDTA and PMSF (at the final concentration of 1 mM each) and concentrating by an Amicon Ultra-15 tube (10 K molecular weight cutoff; Merck, Darmstadt, Germany). Protein concentration was determined using a Qubit Protein Assay kit (Invitrogen, Carlsbad, MA, USA). The obtained *P. insidiosum* CFA (PiCFA) and *B. ranarum* CFA (BrCFA) were stored at −80 °C until use.

### 2.3. Serum Samples

A serum panel was recruited from 3 humans (Serum IDs: HuP1, HuP2, and HuP3) and 3 horses (Serum IDs: HsP1, HsP2, and HsP3) with pythiosis, 5 horses with another infection caused by *B. ranarum* (Serum IDs: HsB1, HsB2, HsB3), *Aspergillus flavus* (Serum IDs: HsC4), and *Actinomyces* species (Serum IDs: HsC5), 4 healthy individuals (Serum IDs: HuC1, HsC1, HsC2, and HsC3), 1 PiCFA-immunized rabbit (Serum ID: RbCFA; from the previous study [24]), and 1 I06 peptide-C-immunized rabbit (Serum ID: RbI06; purchased from Mimotopes, Victoria, Australia) (Table 2). The human sera were obtained from the Department of Pathology, Faculty of Medicine, Ramathibodi Hospital, Mahidol University, Thailand. The animal sera were derived from the Department of Microbiology and Immunology and Equine Clinic, Faculty of Veterinary Medicine, Kasetsart University, Thailand. The diagnosis of each case was confirmed by culture or molecular method [17,22,23,25]. This panel of sera was used for both ICT and WB studies performed against the PiCFA and BrCFA crude extracts and the synthesized I06 protein.

### 2.4. Cell-Free Synthesis of I06 Protein

The I06 protein was synthesized using the method described by Sae-Chew et al. [21]. In short, the gene-specific primers (forward primer I06-1F: ‘5-adaptor-GTCGAGTTCTCTATCACG-3’; reverse primer I06-1R: ‘5-adaptor-CAGCGACTTCTTGTCAGA-3′) were designed and purchased from Bioneer (Daejeon, Korea), according to the manufacturer’s protocol. Although the adaptor sequences were undisclosed by the company (Bioneer, Daejeon, Korea), the I06-1F adaptor sequence contained a start codon (ATG), while the I06-1R adaptor sequence contains a stop codon (TAG). These primers (10 pmol each), together with an ExiProgen ProXpress PCR template kit (Bioneer, Daejeon, Korea), amplified the I06 protein-coding sequence from gDNA of *P. insidiosum* strain Pi–S extracted by the established protocol [26]. The first-step PCR condition comprised the initial 94 °C denaturation (5 min), 30 cycles of 94 °C denaturation (30 s), 58 °C annealing (30 s), and 72 °C elongation (90 s), and the final 72 °C elongation (5 min). After electrophoresis (100 V for 30 min), the 1530 bp amplicon was isolated from a 1.5% agarose gel and purified using an AccuPrep PCR/Gel Purification kit (Bioneer, Daejeon, Korea).

The purified amplicon (10 ng; serving as the second-step PCR template), the ExiProgen ProXpress PCR template kit reagents, the T7 promoter/RBS/6xHistidine-containing upstream cassette (5 ng), the T7 terminator-containing downstream cassette (5 ng), the kit-provided forward (2F) and reverse (2R) primers (10 pmol each), and nuclease-free water were mixed to a 20 μL volume. PCR conditions included the initial 94 °C denaturation (5 min), 30 cycles of 94 °C denaturation (1 min), 48 °C annealing (1 min), 72 °C elongation (90 s), and the final 72 °C elongation (5 min). The purified amplicon (1 μg) was subjected to the cell-free synthesis and purification of the I06 protein using an ExiProgen EC Protein Synthesis kit and an automated cell-free protein synthesis machine (Bioneer, Daejeon, Korea). The protein concentration was assessed using the Protein Assay reagent (Bio-rad, Hercules, CA, USA). The synthesized I06 protein was kept at −20 °C.

### 2.5. Rabbit Anti-I06 Peptide Antibodies and ELISA Testing

**Figure 1:**
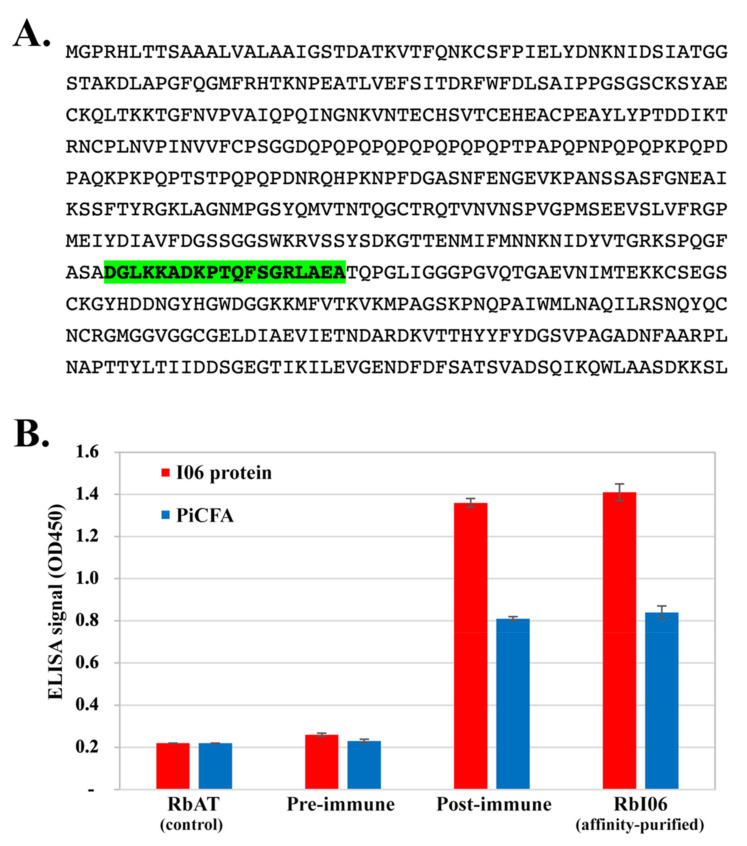
The OPEL-like I06 protein of *P. insidiosum* and rabbit antisera. (**A**) The amino acid sequence of the full-length I06 protein showing the peptide containing a predicted B-cell epitope (namely I06 peptide-C; labeled green); (**B**) ELISA signals (optical density at the 450 nm wavelength) of either the synthesized I06 protein (I06 protein) or *P. insidiosum* crude extract (PiCFA) against rabbit anti-human alpha-tubulin serum (RbAT; served as a control), rabbit pre-immunized serum (pre-immune), rabbit anti-I06 peptide-C serum (post-immune), and affinity-purified anti-I06 peptide-C antibodies (RbI06).

The DNASTAR program (https://www.dnastar.com/) predicted an antigenic peptide (amino acid sequence: ^368^DGLKKADKPTQFSGRLAEA^386^), namely an I06 peptide-C, from the 572-amino acid-long I06 protein sequence (Figure 1A). Rabbit anti-I06 peptide-C serum was synthesized and purchased from Mimotopes (Victoria, Australia). The I06 peptide-C was synthesized (>85% purity), tagged with keyhole limpet hemocyanin (KLH; a carrier protein), and immunized a New Zealand rabbit (7 immunizations in 2 months), according to the company’s protocol. Pre- and post-immunized sera were collected. Rabbit anti-I06 peptide-C antibodies were affinity-purified from the post-immunized antisera, using the I06 peptide-C combined with SulfoLink coupling gel. The rabbit affinity-purified (RbI06), pre-immunized, and remaining post-immunized sera were lyophilized and shipped to Thailand. The lyophilized sera were dissolved in PBS (10 mM Na_2_HPO_4_, 1.8 mM KH_2_PO_4_, 137 mM NaCl, 2.7 mM KCl, pH 7.4) to meet the company’s recommended volume, aliquoted, and stored at −20 °C.

The established ELISA protocol [3] was modified to assess the rabbit anti-I06 protein antibody level. In brief, a 96-well polystyrene plate (Corning, NY, USA) was coated with either the synthesized I06 protein (0.1 µg/well) or PiCFA (5 µg/well) at 4 °C overnight. After washing the unbound proteins 4 times, each well was blocked using PBS with 0.5% bovine serum albumin (250 µL/well) at 37 °C for 60 min. Following another washing step, the RbI06, pre-immunized, and post-immunized sera (each diluted at 1:1000 in PBST-azide (137 mM NaCl, 2.7 mM KCl, 10 mM Na_2_HPO_4_, 1.8 mM KH_2_PO_4_, 0.1% Tween−20, 0.1% Sodium azide)) were added to wells, incubated at 37 °C for 60 min. A rabbit anti-human alpha-tubulin serum (Serum ID: RbAT; Santa Cruz Biotechnology, Dallas, TX, USA) (1:1000 in PBST-azide) was included as a negative control. After washing, the peroxidase-conjugated protein A/G (Bio-Rad, Hercules, CA, USA; 1:100,000 in PBST with 1% bovine serum albumin; 100 µL/well) was added and incubated at 37 °C for 60 min. The plate was washed once again. The 3,3′,5,5′-tetramethylbenzidine and H_2_O_2_ (Thermo Scientific, Waltham, MA, USA) were the in-use chromogenic substrate (100 µL/well). Sulfuric acid (0.5 N; 100 µL/well) stopped the ELISA reaction. An ELISA signal (optical density at the 450 nm wavelength; OD450) was measured. All serum samples were tested in duplicate.

### 2.6. Immunochromatographic Test

**Figure 2:**
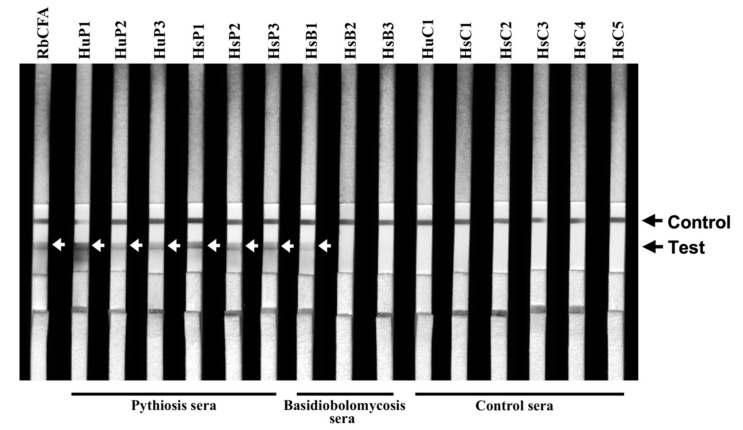
ICT testing against the serum panel. The panel sera are from 3 humans (HuP1–3) and 3 horses (HsP1–3) with pythiosis, 5 horses with another infection caused by *B. ranarum* (HsB1–3), *Aspergillus flavus* (HsC4), and *Actinomyces* species (HsC5), 4 healthy individuals (HuC1, HsC1–3), and 1 *P. insidiosum* CFA-immunized rabbit (RbCFA). Black arrows indicate the control and test lines of the ICT assay. White arrows show the generated test line of each ICT-positive serum sample.

The protein A/G-based ICT assay [2] was used to detect the anti-*P. insidiosum* antibodies in 16 recruited serum samples of humans and animals with or without pythiosis (Serum IDs: RbCFA, HuP1–3, HsP1–3, HsB1–3, HuC1, and HsC1–5) (Table 2). Briefly, an ICT strip was dipped into 100 µL of a diluted serum (1:5000 (animal serum) and 1:10,000 (human serum) in PBS). The antibodies formed a complex with the colloidal gold-conjugated protein A/G and diffused through the ICT strip. The anti-*P. insidiosum* antibodies, if present, bound the pre-blotted PiCFA and generated a visualized signal at the test line (Figure 2). The remaining colloidal gold-protein A/G-antibody complex passed through the test line, captured the pre-blotted rabbit anti-IgG antibodies, and generated a visualized signal at the control line (Figure 2). The ICT result was read within 30 min by experienced personnel. The presence of both the test and control lines was read ICT positive, while the presence of the only control line was read ICT negative. Each tested strip was photocopied using a ChemiDoc MP Imaging System machine (Bio-Rad, Hercules, CA, USA).

### 2.7. Western Blot Analysis

The PiCFA, BrCFA, and the synthesized I06 protein were separated by SDS-PAGE and transferred onto a nitrocellulose membrane (0.45 µm pore size; Bio-Rad, Hercules, CA, USA) using a Power Blotter XL semi-dry transferring machine (Invitrogen, Carlsbad, MA, USA) (setting: 1 Amp for 1 h). Molecular weight markers were run in parallel (Precision Plus Protein Kaleidoscope Standards, Bio-Rad, Hercules, CA, USA). The blotted membrane was incubated with gentle shaking in blocking buffer (5% skim milk in TBS-T buffer (20 mM Tris, 150 mM NaCl, 0.1% Tween 20; pH 7.4)) at 4 °C for overnight. The blotted membrane was incubated with each serum sample (prediluted at 1:1000 in the blocking buffer) (Table 2) at room temperature for 3 h. The membrane was washed with TBS-T 3 times (5 min each), incubated with the horseradish peroxidase-conjugated protein A/G (Thermo Scientific, Waltham, MA, USA) (1:50,000 dilution in blocking buffer) at room temperature for 2 h, and washed again with TBS-T 3 times and PBS 2 times. Finally, the probed membrane was incubated with colorimetric substrate solution (0.03% DAB, 0.05% CoCl_2_, and 0.03% H_2_O_2_ in PBS) to generate WB signals. The processed membrane was photocopied using the ChemiDoc MP Imaging System (Bio-Rad, Hercules, CA, USA).

### 2.8. Statistical Analysis

Sensitivity, specificity, positive predictive value (PPV), and negative predictive value (NPV) of ICT were calculated based on the ICT-tested animals with a definitive diagnosis confirmed by culture or molecular method (Table 1). Sensitivity was the percentage of ICT-positive cases in all pythiosis-confirmed cases, while specificity was the percentage of ICT-negative cases in all non-pythiosis-confirmed cases. PPV was the percentage of true ICT-positive cases in all ICT-positive cases, whereas NPV was the percentage of true ICT-negative cases in all ICT-negative cases.

## 3. Results

### 3.1. Clinical Application of ICT for Serodiagnosis of Animal Pythiosis

During August 2017 and January 2021, a total of 38 animals (including 20 dogs, 15 horses, 2 cats, and 1 elephant) were subjected to ICT testing at the Department of Microbiology and Immunology, Faculty of Veterinary Medicine, Kasetsart University, Bangkok, Thailand (Table 1). Most animals came with a suspected clinical feature of pythiosis, such as a chronic wound, mass or granuloma of the gastrointestinal tract, skin, or spinal cord, and empyema. Seven healthy dogs were checked for the presence of the anti-*P. insidiosum* antibodies because they were in close contact with a known case of pythiosis. Twenty animals (52.6% of all cases) were ICT positive, five of which (four horses and a dog) showed a weakly positive result (Table 1). ICT-negative results (n = 18) were reported in 11 dogs (including all healthy dogs), 4 horses, 2 cats, and the elephant.

Tissue specimens from 27 out of 38 ICT-tested animals (71.1%) were available for fungal culture and molecular assay, comprising 18 ICT-positive and 9 ICT-negative cases (Table 1). The results of the ICT-negative cases showed *Aspergillus flavus* (n = 1), *Microsporum canis* (n = 1), *Nocardia* species (n = 1), dark fungus (n = 1), hyaline septate fungus (n = 2), or no growth (n = 3) (all nine ICT negatives were confirmed to be “true negative” or “non-pythiosis”). On the other hand, the findings of the ICT-positive cases included *P. insidiosum* (n = 13), *B. ranarum* (n = 4), and *Nocardia* species (n = 1) (13 out of 18 ICT positives were confirmed to be “true positive” or “pythiosis”). Either *B. ranarum* (three cases) or *Nocardia* species (one case) was detected in all horses with an ICT-weakly positive result. *B. ranarum* was also isolated from an ICT-positive dog. Based on these 27 animals with a confirmed diagnosis, the ICT performance was calculated to be 100.0% detection sensitivity, 64.3% detection specificity, 72.2% PPV, and 100.0% NPV.

### 3.2. Retesting the ICT False-Positive Sera for Cross-Reactive Antibodies

The panel sera (obtained from three humans and three horses with pythiosis, five horses with another infectious condition, one human and three horses without a recognized disease (healthy), and one PiCFA-immunized rabbit) were tested for cross-reactive antibodies using ICT (Table 2). Three serum samples (HsB1, HsB2, and HsB3) were derived from the known ICT-false positive horses with basidiobolomycosis (Case IDs 06, 12, and 13, respectively; Table 1). The pythiosis (HuP1–3 and HsP1–3) and RbCFA serum samples were tested positive by ICT (Figure 2; Table 2). All control samples (HsB2, HsB3, HsC1–5, and HuC1) were ICT negative, except HsB1, which read weakly positive, as a faint test line was observed (Figure 2).

### 3.3. Western Blot Analyses Reveal Cross-Reactive Antigens of P. insidiosum

**Figure 3:**
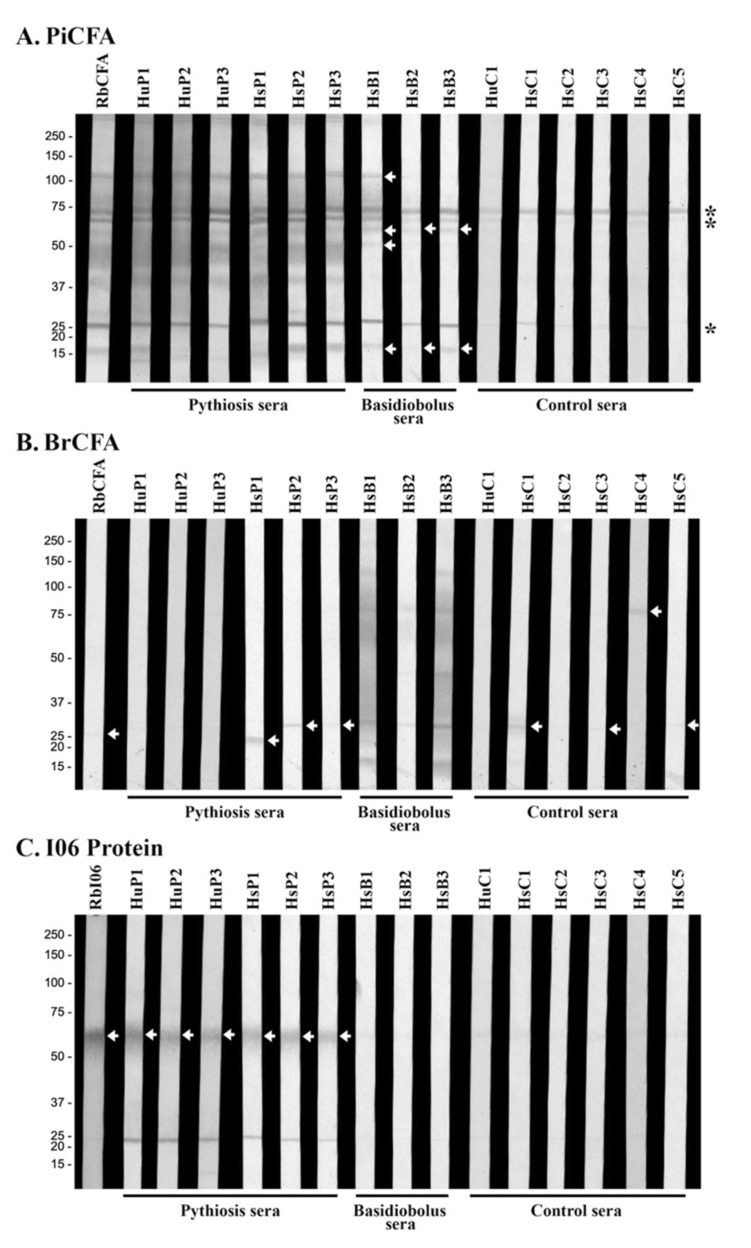
Western blot analyses of (**A**) *P. insidiosum* crude extract (PiCFA), (**B**) *B. ranarum* crude extract (BrCFA), and (**C**) synthesized 55 kDa I06 proteins (I06 protein) against the serum panel. The panel sera are derived from 3 humans (HuP1–3) and 3 horses (HsP1–3) with pythiosis, 5 horses with another infection caused by *B. ranarum* (HsB1–3), *Aspergillus flavus* (HsC4), and *Actinomyces* species (HsC5), 4 healthy individuals (HuC1, HsC1–3), 1 PiCFA-immunized rabbit (RbCFA), and 1 I06 peptide-C-immunized rabbit (RbI06). Background immunoreactive bands are marked with an asterisk. White arrows indicate immunoreactive proteins. Molecular weight markers are shown on the left.

The crude protein extracts of *P. insidiosum* (PiCFA) and *B. ranarum* (BrCFA) were investigated for immunological cross-reactivity. PiCFA and BrCFA were separated by SDS-PAGE, transferred onto a WB membrane, and probed with the same serum panel used for ICT retesting (Table 2). When PiCFA was analyzed, three background proteins (25, 65, and 75 kDa) were immunoreactive to all sera tested, including the control samples from the healthy individuals (Figure 3A). Sera from all six pythiosis subjects (HuP1–3 and HsP1–3) and the PiCFA-immunized rabbit (RbCFA) reacted with multiple proteins of PiCFA (15–120 kDa), including some prominent bands (15, 25, 37, 40–50, 55, 60, 65, 70, 75, and 120 kDa) (Figure 3A). Serum samples (HsB1–3) of three horses with basidiobolomycosis consistently generated WB signals at the 15 and 60 kDa proteins (Figure 3A). Only HsB1 markedly reacted with two additional proteins: 50 and 120 kDa.

No shared immunoreactive protein was observed from the WB analysis of BrCFA against the serum panel (Figure 3B; Table 2). All three basidiobolomycosis sera, especially HsB1 and HsB3, reacted with a broad range of *B. ranarum* proteins, including 15, 30, 60–70, 75, and 120 kDa major bands (Figure 3B). The RbCFA, pythiosis (HyP1–3 and HsP1–3), and control (HuC1 and HsC1–5) serum samples showed no WB signal against most *B. ranarum* proteins, except three faint bands: 20 kDa (for HsP1); 25 kDa (for RbCFA, HsP2, HsP3, HsC1, HsC3, and HsC5); and 75 kDa (for HsC4) (Figure 3B).

### 3.4. Investigation of the Cross-Reactive Antibodies Using the Synthesized I06 Protein

The I06 peptide-C (sequence: ^268^DGLKKADKPTQFSGRLAEA^386^), containing a linear B-cell epitope, was predicted based on the macromolecular structure, motion, and function of the I06 protein (Figure 1A). The I06 peptide-C was synthesized and conjugated with the carrier protein KLH for generating a set of rabbit antisera (i.e., pre-immunized, post-immunized, and affinity-purified (namely RbI06) sera). When using the I06 protein-coated plate, ELISA signals of the post-immunized serum (OD450: 1.36) and RbI06 (OD450: 1.41) were 5.23- and 5.42-fold higher than that of the pre-immunized serum (OD450: 0.26), respectively (Figure 1B). Likewise, when using the PiCFA-coated plate, ELISA signals of the post-immunized serum (OD450: 0.81) and RbI06 (OD450: 0.84) were 3.52- and 3.65-fold higher than that of the pre-immunized serum (OD450: 0.23), respectively (Figure 1B). RbI06 was then used as a positive control in WB analysis of the synthesized I06 protein against the serum panel (Table 2).

The 55 kDa I06 protein (Figure 1A) was generated by cell-free protein synthesis, as described by Sae-Chew et al. [21]. The synthesized I06 protein was separated by SDS-PAGE, transferred onto a WB membrane, and probed with the serum panel (Table 2). The I06 protein strongly reacted to RbI06 and pythiosis sera (HyP1–3 and HsP1–3) but generated no WB signal against basidiobolomycosis (HsB1–3) and control (HuC1 and HsC1–5) sera (Figure 3C).

## 4. Discussion

Intaramat et al. developed ICT, a rapid and user-friendly test, for the serodiagnosis of pythiosis in humans and animals [2]. Their assay appeared to be sensitive (90.6%) and specific (100.0%), based on the evaluation against serum samples from 85 proven cases of pythiosis and 143 control subjects with, for example, aspergillosis, zygomycosis, candidiasis, cryptococcosis, histoplasmosis, blastomycosis, protothecosis, sporotrichosis, *Lagenidium giganteum* infection, and *Paralagenidium karlingii* infection [2]. In this study, when 38 animals with suspected pythiosis were tested with ICT, 20 (52.6%) and 18 (47.4%) were reported as positive and negative, respectively (Table 1). Based on tissue sample availability, the culture and molecular test can confirm the diagnosis in 27 animals, comprising 18 ICT-positive and 9 ICT-negative cases (Table 1). Focusing on these 27 animals with a definitive diagnosis, ICT exhibited 100% detection sensitivity, as all confirmed pythiosis cases (n = 13) were ICT-positive, and 100% NPV, as all ICT-negative cases (n = 9) were confirmed as non-pythiosis. However, the assay showed a limited detection specificity (64.3%), as 5 out of 14 confirmed non-pythiosis cases were ICT-positive, and a poor PPV (72.2%), as 5 out of 18 ICT-positive cases were confirmed as non-pythiosis. Therefore, our findings ensured the high detection sensitivity but raised a concern on the critically low detection specificity of the current ICT assay [2]. Nevertheless, ICT is still helpful for screening pythiosis in a clinically suspected case, although a positive result needs confirmation by culture or molecular method. For example, two ICT-positive animals (i.e., case IDs 32 and 38; Table 1) needed to be confirmed “pythiosis” by a more specific test, as the antibodies against another pathogen might generate a false-positive ICT result.

As shown here, basidiobolomycosis can be serologically misdiagnosed as pythiosis (Table 1). The antigens currently used in ICT, WB, and other assays for diagnosing pythiosis are the crude extract of *P. insidiosum* (i.e., PiCFA) [1,2,3,4,5,6,7,8,9,13]. Such an antigen source contains various undefined proteins, some of which may share a protein or carbohydrate epitope with another microorganism, leading to immunological cross-reactivity. For example, the anti-*B. ranarum* antibodies (presented in the basidiobolomycosis cases) might bind some *P. insidiosum* proteins (used in the assay development), contributing to the ICT false-positive results (Table 1). We addressed this hypothesis by analyzing the serum panel (Table 2) against PiCFA (Figure 3A) and BrCFA (Figure 3B). Three WB bands (25, 65, and 75 kD) were consistently reacted with by all panel sera and considered non-specific background signals (Figure 3A). All basidiobolomycosis sera (HsB1–3), but no other controls, can react several extra proteins of PiCFA (15, 50, 60, and 120 kDa), demonstrating significant cross-reactions between basidiobolomycosis sera and *P. insidiosum* proteins (Figure 3A). However, only one of the three WB-positive basidiobolomycosis sera (HsB1; which had the highest WB signal) was retested as positive by ICT (Figure 2), partly due to the fact that these sera were repeatedly frozen and thawed and the optimized serum dilution of WB (1:1000) was lower than that of ICT (1:5000). In addition, the different optimal serum dilutions of ICT and WB analyses against the same antigens (i.e., PiCFA) could also explain why the background WB signals (i.e., 25, 65, and 75 kD bands; Figure 3A) were not detected by ICT (Figure 2). Compared to WB, the markedly higher serum dilution (1:5000; reflecting a lower level of antibodies) for ICT testing against all panel sera can eliminate the signal of the non-specific background WB bands (as the control sera provided no test line; Figure 2). Regarding BrCFA, this antigen source prominently reacted to the basidiobolomycosis sera (HsB1–3) and showed a few faint WB bands (20, 25, and 75 kDa) against several pythiosis and control sera (Figure 3B). We noted that the rabbit antiserum RbCFA, specifically raised against PiCFA (Figure 3A), weakly reacted only the 25 kDa band of BrCFA (Figure 3B). These findings suggested that the immunological cross-reactivity of pythiosis sera and BrCFA was not as solid and consistent as the basidiobolomycosis sera and PiCFA.

The false-positive ICT (Figure 2) and WB (Figure 3A) results may also occur due to double infections of *P. insidiosum* and *B. ranarum*. The I06 protein, present in *P. insidiosum* but no other medically important fungi [21], was subject to WB analysis against the serum panel. Only the pythiosis sera and RbI06 (positive control), but none of basidiobolomycosis and other negative control sera (Table 2), significantly generated WB signals against the synthesized I06 protein (Figure 3C). This finding indicated that the false-positive serology was solely due to the cross-reactivity of anti-*B. ranarum* antibodies and *P. insidiosum* antigens, not double infections.

Taxonomies of *P. insidiosum* (a fungus-like oomycete organism [10,12,27]) and *B. ranarum* (a true fungus in the order Entomophthorales [28,29]) are distinctly different. However, both pathogens infect humans and various animals and could lead to indistinguishable clinical presentations [9,10,11,12,27,28,29,30,31]. In addition, the histopathological features of *P. insidiosum* (i.e., broad hyphae) are similar to *B. ranarum*, even causing delayed diagnosis and treatment of the affected patient. Thus, the specificity of ICT for rapidly detecting anti-*P. insidiosum* antibodies needs to improve. The multi-antigen-containing crude extract, used to develop a serological test, needs to be replaced with a refined *P. insidiosum*-specific immunoreactive protein. The publicly available genome data of *P. insidiosum* is an invaluable resource in the search for such a protein. Our study showed that the I06 protein, uniquely identified in the *P. insidiosum* genome [32,33,34], could be an excellent protein candidate for developing a pythiosis-specific serological test (i.e., ICT and ELISA). An extensive set of pythiosis and control sera (including those from basidiobolomycosis patients) will be required to develop and evaluate such a serological test.

In conclusion, pythiosis has been increasingly diagnosed worldwide. Serological tests bypass the experience-requiring and time-consuming microbiological techniques (i.e., culture and molecular method) to provide a rapid diagnosis of pythiosis, leading to prompt treatment and better prognosis. ICT appeared to be a quick, user-friendly, and efficient assay for pythiosis. However, we detected ICT false-positive results in animals with basidiobolomycosis due to the cross-reactivity of anti-*B. ranarum* antibodies (in the sera) to the 15, 50, 60, and 120 kDa proteins of *P. insidiosum* (in the developed assay). Nevertheless, ICT is still helpful for screening a suspected pythiosis case, in which a positive test requires confirmation by culture or molecular method. The detection specificity of serological assays for pythiosis needs improvement, and thus the crude extract containing multispecies antigens needs replacement with a refined *P. insidiosum*-specific protein. Based on this preliminary study, we proposed that the synthesized I06 protein is an excellent candidate for developing a sensitive and specific serodiagnostic test for pythiosis.

## Figures and Tables

**Table 1:** Laboratory test results of 38 animals with suspected pythiosis during the period between August 2018 and January 2021.

Year	Case ID	Affected Animal	Clinical Diagnosis	Diagnostic Method	
				ICT for Pythiosis	Culture/Molecular Assay
2017	01	Dog	Pythiosis	Positive	*Pythium insidiosum*
2017	02	Horse	Pythiosis	Positive	*Pythium insidiosum*
2017	03	Horse	Pythiosis	Positive	*Pythium insidiosum*
2017	04	Horse	Pythiosis	Positive	*Pythium insidiosum*
2017	05	Horse	Pythiosis	Positive	*Pythium insidiosum*
2017	06	Horse	Fungal granuloma	Weakly positive *	*Basidiobolus ranarum*
2018	07	Horse	Guttural pouch empyema	(Negative)	*Aspergillus flavus*
2018	08	Horse	Chronic wound	(Negative)	Dark fungus
2018	09	Horse	Pythiosis	Positive	*Pythium insidiosum*
2018	10	Horse	Pythiosis	Positive	*Pythium insidiosum*
2018	11	Horse	Habronemiasis	Weakly positive *	*Nocardia* species
2018	12	Horse	Chronic wound	Weakly positive *	*Basidiobolus ranarum*
2018	13	Horse	Chronic wound	Weakly positive *	*Basidiobolus ranarum*
2019	14	Cat	Spinal cord granuloma	(Negative)	*Microsporum canis*
2019	15	Dog	Cutaneous granuloma	(Negative)	Hyaline septate fungus
2019	16	Dog	Chronic wound	(Negative)	No growth
2019	17	Dog	Healthy	(Negative)	None
2019	18	Dog	Healthy	(Negative)	None
2019	19	Dog	Healthy	(Negative)	None
2019	20	Dog	Healthy	(Negative)	None
2019	21	Dog	Healthy	(Negative)	None
2019	22	Dog	Healthy	(Negative)	None
2019	23	Dog	Healthy	(Negative)	None
2019	24	Dog	GI granuloma	Positive	*Pythium insidiosum*
2019	25	Dog	GI granuloma	Positive	*Pythium insidiosum*
2019	26	Dog	Chronic wound	Weakly positive	*Pythium insidiosum*
2019	27	Horse	Chronic wound	(Negative)	*Nocardia* species
2019	28	Horse	Chronic wound	(Negative)	None
2020	29	Cat	Chronic wound	(Negative)	Hyaline septate fungus
2020	30	Dog	Chronic wound	(Negative)	No growth
2020	31	Dog	Chronic wound	(Negative)	No growth
2020	32	Dog	GI granuloma	Positive	None
2020	33	Dog	Cutaneous granuloma	Positive	*Pythium insidiosum*
2020	34	Dog	Pythiosis	Positive	*Pythium insidiosum*
2020	35	Dog	GI infection	Positive *	*Basidiobolus ranarum*
2020	36	Elephant	Unknown	(Negative)	None
2020	37	Horse	Pythiosis	Positive	*Pythium insidiosum*
2021	38	Dog	Gastric mass	Positive	None

**Footnote**: * False positive result.

**Table 2:** A panel of pythiosis, basidiobolomycosis, and control sera used in this study and immunochromatographic test (ICT) results.

Serum ID	Host	Cause of Infection	ICT Result
HuP1	Human	*Pythium insidiosum*	Positive
HuP2	Human	*Pythium insidiosum*	Positive
HuP3	Human	*Pythium insidiosum*	Positive
HsP1	Horse	*Pythium insidiosum*	Positive
HsP2	Horse	*Pythium insidiosum*	Positive
HsP3	Horse	*Pythium insidiosum*	Positive
HsB1	Horse	*Basidiobolus ranarum*	Weakly positive
HsB2	Horse	*Basidiobolus ranarum*	(Negative)
HsB3	Horse	*Basidiobolus ranarum*	(Negative)
HuC1	Human	None (Healthy)	(Negative)
HsC1	Horse	None (Healthy)	(Negative)
HsC2	Horse	None (Healthy)	(Negative)
HsC3	Horse	None (Healthy)	(Negative)
HsC4	Horse	*Aspergillus flavus*	(Negative)
HsC5	Horse	*Actinomyces* species	(Negative)
RbCFA	Rabbit	None (anti-PiCFA serum)	Positive
RbI06	Rabbit	None (anti-I06 peptide serum)	None
RbAT	Rabbit	None (anti-alpha tubulin serum)	None
